# 

**Published:** 1908-05-09

**Authors:** 


					Mas 9, 1908. THE HOSPITAL.
CONTENTS.
OMlirv.
Opium and Alcohol
137
Qenp : "'UUUOl
Partmen?CtltIoners and tlie ?ut"Patient De"
138
Annotations^
^HealthS0 diseases?Preservatives in Food?Altitude and
139
Opinion and Movement
140
H?8plta,cunlcs-
a'auavfu una xuovemeni
rpT -J," ?
I0'?Sy and Treatment of Nocturnal Enuresis.
By Jas.
1, M.A., M.D..
143
Abscess.
By William Turner, M.S., M.B., F.It.C.S.
Medicine- SS"
By William Turner, M.S., M.B., F.Il.C.S. ...
of Carcinoma Ventriculi
^return Sundaionm in flip flnrfi of tlm Oninm Habit
?'aSn?sis of Carcinoma Ventrieuli
147
^Olnfr
Coini.vY ?"""?guusis or carcinoma ventricuii
1B*_ JJctu*p Sundaicum in the Cure of the Opium Habit
148
^oint. j oundaicui
Tho cfn Surgery-
^um bundaicum in the Cure of the Opium Habit
Thoc
Anenr r^1Ca^ Significance of Vomiting
149
Aneurysm ? cance or vomimiS
150
Obstetrics?
Obstetrics at Queen Charlotte's Hospital ...
151
Dermatology -
The Diagnosis of Ringworm of the Scalp
152
The General Practitioner's Column-
Local Treatment of the Commoner Acute Throat Affections ...
153
Filing of Newspaper Cuttings ...
154
Hospital Administration?
South London Practitioners and the Removal of King's
College Hospital _
155
Glasgow Maternity and Women's Hospital ...
157
News and Coming Events
159
Modern Gas Cooking Stoves
160
Nursing Administration?
The Bedding and Linen Department...
161
Editor's I>etter-Box-
Cow's Milk and Kickets ...
162
Tbe Graduates' XtXedlcal Schools

				

